# Preoperative anemia and deep vein thrombosis in patients with perioperative bone trauma: a cohort study

**DOI:** 10.1186/s12891-022-05869-7

**Published:** 2022-10-10

**Authors:** Hui Zhang, Linqin Wu, Bo Cheng

**Affiliations:** Department of Anesthesiology, The First Affiliated Hospital of Chongqing Medical University, Yuzhong District, Chongqing, 400000 China

**Keywords:** Preoperative anemia, Deep vein thrombosis, Propensity score matching, Perioperative

## Abstract

**Background:**

In current active prevention (including physical and drug prevention), the incidence of perioperative deep vein thrombosis (DVT) of the lower extremities remains high in patients with bone trauma. Risk factors need to be further optimized, and high-risk patients must be identified early. Preoperative comorbidities, especially preoperative anemia, and DVT in patients with perioperative bone trauma are not clear. The purpose of this study was to explore the causal relationship between preoperative anemia and DVT in patients with perioperative bone trauma, and further reduce the incidence of DVT in patients with bone trauma.

**Objectives:**

To analyze the relationship between preoperative anemia and perioperative DVT in patients with femoral and pelvic fractures and provide a reference for the optimization of risk factors for DVT.

**Methods:**

The clinical data of 1049 patients with femoral and pelvic fractures who received surgical treatment from May 2018 to June 2021 were retrospectively analyzed. Propensity score matching (PSM) was performed for the covariates of DVT. Modified Poisson regression was used to analyze the relationship between preoperative anemia and DVT.

**Results:**

After matching 1:1 propensity scores in 1049 patients included in this study, there were 258 patients in the anemic and non-anemic groups. Preoperative anemia was statistically significant for the formation of DVT in patients with perioperative bone trauma (*P* = 0.000, RR = 1.567 [95% CI 1.217–2.017]). This conclusion remained true after PSM (*P* = 0.009, RR = 1.500 [95% CI 1.105–2.036]). Preoperative anemia has some predictive value for perioperative DVT, with DVT-associated preoperative anemia thresholds of 125 g/L and area under the receiver operating characteristic curve of 0.5877 (95% CI 0.5345 to 0.6408). On this basis, sensitivity and specificity were 89.2 and 30.3%, respectively, with a Youden index of 0.195. In addition, we conducted an E-value determination of the propensity score; the E-value analysis showed robustness to unmeasured confounding.

**Conclusions:**

Preoperative anemia is highly correlated with perioperative DVT in patients with bone trauma, which is the cause of perioperative DVT in these patients.

## Introduction

Venous thromboembolism (VTE), including deep vein thrombosis (DVT) and pulmonary embolism (PE), is a common complication and cause of death in patients with bone trauma [1]. The incidence of VTE in orthopedics, especially in patients with bone trauma, is very high. Clinical preventive measures include anticoagulation and physiotherapy, such as lower extremity barometric pressure. Commonly used anticoagulant regimens include: 1) anticoagulant therapy based on the coagulation cascade, such as heparin and rivaroxaban; 2) antiplatelet therapy based on the platelet pathway, such as aspirin. Under the current active prevention, there has been a certain degree of decline in perioperative DVT, but the incidence remains high [2–4]. Postoperative DVT and femoral neck fractures have been reported to be as high as 32 and 56%, respectively [5].

From the perspective of pathogenesis, high hematocrit promotes blood stasis, which is the basic condition for the formation of DVT. However, some studies have confirmed that preoperative anemia is also a risk factor for DVT [5–8]. The mechanism of thrombosis formation under the low hematocrit of anemia is still unclear. Some studies have reported that blood transfusion has a certain influence on the formation of DVT [7, 9–13]. Therefore, we hypothesized that DVT formation based on the red blood cell pathway may be a pathogenic factor for DVT formation in patients with bone trauma. Therefore, we assumed that preoperative anemia is a risk factor for perioperative DVT patients with bone trauma, for verification.

VTE is associated with endothelial dysfunction and blood stasis that result in the formation of fibrin- and red blood cell (RBC)-rich (“red”) clots. From the perspective of pathogenesis, high hematocrit promotes blood stasis, the blood viscosity increases with the increase of hematocrit [6, 7]. Such increases in blood viscosity may promote platelet margination and have physical effects on the interaction between platelets and the blood vessel walls, which have a physical effect on hemostasis and thrombosis. Some studies have reported a 1.5-fold increased risk of first VTE in patients with high versus low hematocrit [8]. However, clinical and epidemiological studies have associated quantitative and qualitative abnormalities in RBCs, including altered hematocrit, perioperative anemia, sickle cell disease, thalassemia, hemolytic anemias, and malaria, with both arterial and venous thrombosis [9–14]. Regardless of hematocrit changes, hemolytic anemia, and perioperative anemia, these all involve lesions or destruction of red blood cells. Therefore, DVT formation based on red blood cell pathway may be a potential pathogenic pathway. Among them, preoperative anemia further increases the rate of perioperative blood transfusion, and previous studies have also pointed out that blood transfusion has a certain effect on venous thrombosis [13, 15–17].

These findings suggest that RBCs may contribute to thrombosis pathophysiology and reveal potential strategies for therapeutically targeting RBCs to reduce thrombosis. The mechanism of thrombosis formation under the low hematocrit of anemia is still unclear. Therefore, we hypothesized that DVT formation based on the red blood cell pathway may be a pathogenic factor for DVT formation in patients with bone trauma. And we assumed that preoperative anemia is a risk factor for perioperative DVT patients with bone trauma, for verification.

## Materials and methods

### Research participants

We conducted retrospective analysis of patients undergoing bone trauma surgery in our hospital from May 2018 to June 2021.Inclusion criteria: (1) Traumatic fractures; (2) Age > 18 years; (3) Definitive diagnosis of femur and pelvic fractures and surgical treatment; (4) Complete medical records. Exclusion criteria: (1) complicated by hematologic diseases or coagulation dysfunction; (2) long-term history of taking anticoagulant drugs; (3) pregnancy; (4) patients with severe diseases of important organs such as liver, kidney, heart, and brain that cannot tolerate surgery; (5) vascular surgery; (6) previous deep vein thrombosis. This study has been approved by the Ethics Committee of the First Affiliated Hospital of Chongqing Medical University (16-06-2021, approval no.2021–235) and registered with the China Clinical Trials Registration Center (01-08-2021, ChiCTR2100049356).

There were 1049 patients recruited in the study period.154 patients did not have ultrasound after surgery, the other 194 patients had preoperative thrombosis and were thus not included in the study. Finally, a total of 701 patients remained for study analysis, including 272 men and 429 women.

### Research methods

The patient’s clinical data were collected, and patients were divided into a non-DVT group and a DVT group, according to the results of preoperative and postoperative double lower extremity deep vein Doppler ultrasound. The DVT group was defined as new or progressive thrombus after surgery. Cases were divided into anemic (exposure group) and non-anemic groups (control group), according to the concentration of hemoglobin in the routine preoperative blood workup, which was the last tests before surgery. Anemia is defined as ≤120 g/L for men and ≤ 110 g/L for women [11, 18]. After adjusting for confounding factors in the two groups after propensity score matching (PSM), the relationship between preoperative anemia and perioperative DVT was analyzed.

### Data collection

Patients’ clinical data were collected from the electronic medical record system and surgical anesthesia system. Preoperative and postoperative pulsed Doppler ultrasonography on both lower limbs was performed using a C5–1 linear probe and an IU22 system (Philips ATL, Bothwell, WA, USA). Diagnostic criteria for DVT included venous incompressibility, intravascular filling defects, and loss of Doppler signal. Common femoral vein, superficial femoral vein and deep femoral vein, popliteal vein, anterior tibial vein, peroneal vein, and intermuscular vein thrombosis were routinely scanned in both lower extremities. A proximal thrombus is defined as a thrombus located in the popliteal, femoral, or iliac veins. Distal thrombus is defined as calf veins (excluding popliteal veins) below the knee, confined to calf veins (peroneal, anterior and posterior tibial, and intermuscular veins). Mixed thrombus was defined as the presence of both distal and proximal thrombi. The development or progression of perioperative DVT was the end point. The primary variable in this study was preoperative anemia.

### Statistical methods

SPSS 23.0 (IBM, Armonk, NY, USA) software and Free Statistics software version 1.5 was used for statistical analysis. An independent samples *t*-test was used for measurement data conforming to a normal distribution. The results are expressed as mean ± standard deviation (x ± s). Non-normally distributed measurement data were tested using the Mann–Whitney U test [19]. The results were expressed as median (quartile) [M (Q1, Q3)]. Enumeration data were analyzed with the chi-square test or Fisher’s exact test. The results are expressed as percentage (%). All statistical tests were two-sided, and *P* < 0.05 was considered statistically significant [20].

To control the influence of confounding factors, PSM was used to build a logistic model, with preoperative anemia as the dependent variable and DVT-related variables as the covariates. 0.02 was used as the caliper value to match the anemic group and non-anemic group at a ratio of 1:1. Covariate variables that may be associated with DVT are as follows: general information such as sex, age, body mass index (BMI), smoking history, alcohol consumption history, and American Society of Anesthesiologists (ASA) classification; comorbidities including cerebral infarction, diabetes, hypertension, coronary heart disease, hyperlipidemia, pulmonary diseases, hepatic diseases, renal diseases, malignancy, and hypoproteinemia; surgery, trauma (time from trauma to surgery, duration of surgery), anticoagulation, and whether blood transfusion was available; and test indicators including preoperative activated partial thromboplastin time (APTT) level, preoperative prothrombin time (PT) level, preoperative white blood cell (WBC) count, preoperative platelet (PLT) count, serum creatinine (Crea) level and preoperative neutrophil percentage (NEUT%). The caliper value was set to take 20% standard deviation of the two sets of propensity scores. The matching effect of PSM was evaluated using the standardized difference method, and the standardized difference value (d) was calculated. If d < 0.1, the matching effect was judged to be good.

The receiver operating characteristic (ROC) curve was further constructed to analyze the predictive significance of the preoperative hemoglobin concentration on the occurrence of perioperative DVT in patients with bone trauma, and the preoperative hemoglobin threshold and its specificity and sensitivity as a predictive factor were calculated.

### Sensitivity analysis

To determine whether the results were sensitive to the matching method, we performed an additional sensitivity analysis using the full queue. First, multivariate Poisson regression was performed after adjusting the covariates associated with DVT to assess the correlation. Second, we calculated the relationship between adjusted perioperative DVT occurrence in patients with preoperative anemia and non-anemia using different models of propensity scores. Finally, we explored the potential for unmeasured confounding between preoperative anemia and perioperative DVT by calculating the E-value. The E-value quantifies the required magnitude of an unmeasured confounder that could negate the observed association between preoperative anemia and perioperative DVT.

## Results

**Fig. 1:**
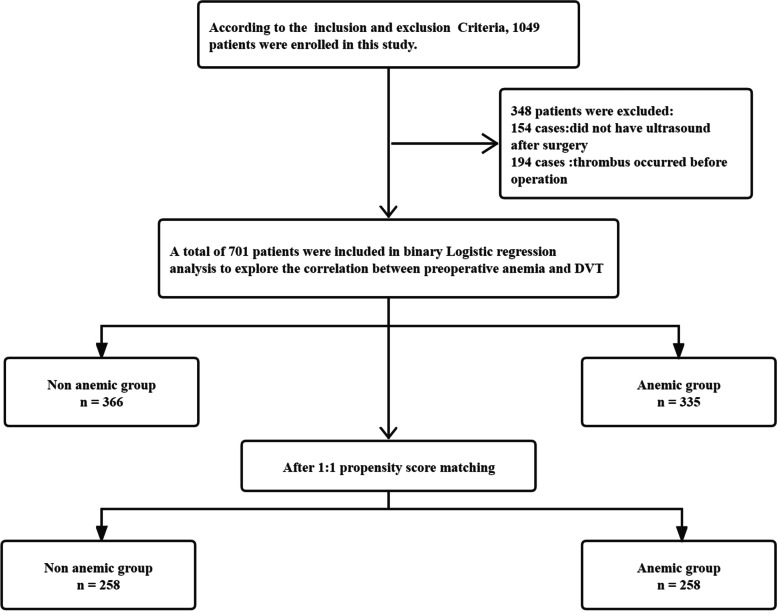
Research flow chart. Inclusion criteria: (1) Traumatic fractures; (2) Age > 18 years; (3) Definitely diagnosis of femur and pelvic and surgical treatment; (4) Complete medical records. Exclusion criteria: (1) complicated by hematologic diseases or coagulation dysfunction; (2) long term history of taking anticoagulant drugs; (3) pregnancy; (4) patients with severe diseases of important organs such as liver, kidney, heart, and brain that cannot tolerate surgery; (5) vascular surgery; (6) previous deep vein thrombosis. Preoperative anemia was taken as the dependent variable, DVT-related variable as the covariable, and 0.02 as the caliper value. Matching was conducted in a ratio of 1:1. DVT means deep vein thrombosis

From May 2018 to June 2021, data were collected for a total of 1049 patients with femur and pelvic fracture surgery at our hospital. In retrospective follow-up, we found that 154 patients did not have ultrasound after surgery (14.68%), and 194 patients had preoperative thrombosis (18.49%). Comparing the two groups of patients who had postoperative ultrasound (895 cases) and those who did not have ultrasound after surgery (154 cases), there was no statistical significance in terms of general characteristics, underlying diseases, comorbidities, or anticoagulation. (*P* > 0.05), which showed that the distribution of the two groups was balanced. This also proved that it was not because the patient had many underlying diseases that ultrasound was performed after surgery (Table 1). New or progressive thrombosis was considered an endpoint event, so we continued to exclude 194 patients with DVT diagnosed with preoperative ultrasound and finally included a total of 701 patients. The inclusion process for the study is presented in Fig. 1. A total of 701 patients were enrolled in this study, including 272 men and 429 women, with a median age of 69.6 ± 19.1 years (20–112 years). Taking 0.02 as the caliper value, 25 factors were used as covariates, including sex, BMI, smoking history, alcohol consumption history, diabetes, hypertension, coronary heart disease, hyperlipidemia, liver disease, kidney disease, lung disease, malignancy, hypoproteinemia, ASA classification, and various test indicators were matched 1:1 between the anemic group and non-anemic group. Finally, 258 pairs of cases were successfully matched in each group.

**Table 1:** Baseline characteristics of participants with and without postoperative ultrasound

covariate	no postoperative ultrasound	postoperative ultrasound	X^**2**^	***P*** value
	***n*** = 154	***n*** = 895		
**Gender, no.(%)**				
Male	69 (44.81)	354 (39.55)	1.506	0.22
Female	85 (55.19)	541 (60.45)		
**Drinking history, no.(%)**				
No	116 (75.32)	69 (77.09)	0.231	0.631
Yes	38 (24.68)	205 (22.91)		
**Smoking history, no.(%)**				
No	125 (81.17)	722 (80.67)	0.021	0.885
Yes	29 (18.83)	173 (19.33)		
**BMI (kg/m**^**2**^**), no.(%)**				
≤ 18	12 (7.79)	108 (12.17)	2.832	0.243
18–25	118 (76.62)	631 (70.50)		
>25	24 (15.59)	148 (16.53)		
**Cerebral infarction, no.(%)**				
No	137 (88.96)	774 (86.48)	0.708	0.4
Yes	17 (11.04)	121 (13.52)		
**Diabetes, no.(%)**				
No	121 (78.57)	728 (81.34)	0.653	0.419
Yes	33 (21.43)	167 (18.66)		
**Coronary heart disease, no.(%)**				
No	137 (88.96)	798 (89.16)	0.005	0.941
Yes	17 (11.04)	97 (10.84)		
**Hypertension, no.(%)**				
No	101 (65.58)	613 (68.49)	0.511	0.475
Yes	53 (34.42)	282 (31.51)		
**Hyperlipidemia, no.(%)**				
No	145 (94.16)	859 (95.98)	1.062	0.303
Yes	9 (5.84)	36 (4.02)		
**Pulmonary diseases, no.(%)**				
No	145 (94.16)	774 (86.48)	7.13	0.008
Yes	9 (5.84)	121 (13.52)		
**Hepatic diseases**				
No	135 (87.66)	777 (86.82)	0.083	0.773
Yes	19 (12.34)	118 (13.18)		
**Renal diseases, no.(%)**				
Yes	144 (93.51)	791 (88.38)	0	0.983
No	10 (6.49)	104 (11.62)		
**Malignant tumor, no.(%)**				
No	145 (94.16)	825 (92.18)	0.738	0.39
Yes	9 (5.84)	70 (7.82)		
**ASA classification, no.(%)**				
≤ 2	58 (37.66)	208 (23.24)	14.439	<0.001
>2	96 (62.34)	687 (76.76)		
**Lung infection, no.(%)**				
No	142 (92.21)	797 (89.05)	1.396	0.237
Yes	12 (7.79)	98 (10.95)		
**Osteoporosis, no.(%)**				
No	100 (64.94)	567 (63.35)	0.142	0.706
Yes	54 (35.06)	328 (36.65)		
**Chronic obstructive pulmonary disease, no.(%)**				
No	141 (91.56%)	779 (87.04)	2.488	0.115
Yes	13 (8.44)	116 (13.96)		
**Anticoagulation, no.(%)**				
No	11 (7.14)	37 (4.13)	2.724	0.099
yes	143 (92.06)	858 (95.07%)

### Comparison of DVT correlative covariates between groups before and after PSM

Before PSM, there were 366 cases (52.21%) in the non-anemic group and 335 cases (47.79%) in the anemic group. There were no significant differences between the two groups in terms of sex, age, smoking history, drinking history, BMI, lung disease, hypertension, coronary heart disease, diabetes, kidney disease, malignancy, and anticoagulation (*P* > 0.05). However, there were statistically significant differences between the groups in ASA grade (*P* = 0.047), hyperlipidemia (*P* = 0.03), liver disease (*P* = 0.033), hypoproteinemia (*P* = 0.000), operation time (*P* = 0.023), time from trauma to surgery (*P* = 0.000), whether blood transfusion was received (*P* = 0.011), preoperative white blood cell (WBC) count, (*P* = 0.005), and preoperative prothrombin time (PT) level (*P* = 0.002).

After caliper matching of data between groups using the PSM method, 258 cases were found in each group. The above 25 covariates related to DVT were not significantly different between the two groups (*P* > 0.05). This indicated that the data of the groups were balanced and comparable, as shown in Table 2.

**Table 2:** Distribution characteristics of covariates in patients who are anemic and non-anemic patients before and after propensity score matching

covariate	Before matching				After matching			
	Non anemia	Anemia	X2/t/Z	***p*** value	Non anemia	Anemia	X2/t/Z	***p*** value
	***N*** = 366	***N*** = 335			***N*** = 258	***N*** = 258		
***General information, complications***								
**Gender, no.(%)**								
Male	138 (37.70)	134 (40.00%)	0.388	0.533	96 (37.21%)	94 (38.43%)	0.033	0.855
Female	228 (62.30)	201 (60.00%)			162 (62.79%)	164 (63.57%)		
**Age-group(y), no.(%)**								
≤ 60	97 (26.50%)	104 (31.04%)	5.398	0.067	72 (27.91%)	80 (31.01%)	4.275	0.118
60–70	68 (18.58)	42 (12.54%)			49 (18.99%)	32 (12.40%)		
>70	201 (54.92%)	189 (56.42%)			137 (53.10%)	146 (56.59%)		
**BMI (Kg/m**^**2**^**), no.(%)**								
≤ 18	39 (10.66%)	52 (15.52%)	4.898	0.086	34 (13.18%)	40 (15.50%)	0.634	0.728
18–25	260 (71.04%)	235 (70.15%)			185 (71.71%)	178 (68.99%)		
>25	67 (18.30%)	48 (14.33%)			39 (15.12%)	40 (15.50%)		
**ASA classification, no.(%)**								
≤ 2	100 (27.32%)	70 (20.90%)	3.933	0.047	61 (23.64%)	60 (23.26%)	0.011	0.917
>2	266 (72.68%)	265 (79.10%)			197 (76.36%)	198 (76.74%)		
**Smoking history, no.(%)**								
No	283 (77.32%)	265 (79.10%)	0.326	0.568	211 (81.78%)	206 (79.84%)	0.312	0.576
Yes	83 (22.68%)	70 (20.90%)			47 (18.22%)	52 (20.16%)		
**Drinking history, no.(%)**								
No	302 (82.51%)	274 (81.79%)	0.062	0.803	216 (83.72%)	216 (83.72%)	0	1
Yes	64 (17.49%)	61 (18.21%)			42 (16.28%)	42 (16.28%)		
**Cerebral infarction, no.(%)**								
No	318 (86.89%)	285 (85.1%)	0.477	0.49	222 (86.05%)	221 (85.66%)	0.016	0.899
Yes	48 (13.11%)	50 (14.9%)			36 (13.95%)	37 (14.34%)		
**Diabetes, no.(%)**								
No	288 (78.7%)	280 (83.58%)	2.725	0.099	214 (82.95%)	212 (82.17%)	0.054	0.817
Yes	78 (21.3%)	55 (16.42%)			44 (17.05%)	46 (17.83%)		
**Coronary heart disease, no.(%)**								
No	327 (89.34%)	292 (87.16%)	0.805	0.37	232 (89.92%)	226 (87.60%)	0.699	0.403
Yes	39 (10.66%)	43 (12.84%)			26 (10.08%)	32 (2.40%)		
**Hypertension, no.(%)**								
No	238 (65.02%)	234 (69.85%)	1.85	0.174	181 (70.16%)	175 (67.83%)	0.326	0.568
Yes	128 (35.98%)	101 (30.15%)			72 (29.84%)	83 (32.17%)		
**Hyperlipidemia, no.(%)**								
No	347 (94.81%)	328 (97.91%)	4.712	0.03	252 (97.67%)	251 (97.29%)	0.079	0.797
Yes	19 (5.19%)	7 (2.09%)			6 (2.33%)	7 (2.71%)		
**Pulmonary diseases, no.(%)**								
No	325 (88.80%)	288 (85.97%)	1.274	0.259	223 (86.43%)	226 (97.29%)	0.154	0.694
Yes	41 (11.20%)	47 (14.03%)			35 (13.57%)	32 (12.40%)		
**Hepatic diseases, no.(%)**								
No	309 (84.43%)	301 (89.85%)	4.556	0.033	236 (91.47%)	232 (89.92%)	0.368	0.544
Yes	57 (15.57%)	34 (10.15%)			22 (8.53%)	26 (10.08%)		
**Renal diseases, no.(%)**								
No	328 (89.62%)	289 (86.27%)	1.86	0.173	229 (88.76%)	228 (88.37%)	0.019	0.89
Yes	38 (10.38%)	46 (13.73%)			29 (11.24%)	30 (11.63%)		
**Malignant tumor, no.(%)**								
No	339 (92.62%)	311 (92.84%)	0.012	0.914	235 (91.09%)	240 (93.02%)	0.662	0.416
Yes	27 (7.38%)	24 (7.16%)			23 (8.91%)	18 (6.98%)		
**Hypoproteinemia, no.(%)**								
No	307 (83.88%)	222 (66.27%)	29.298	<0.001	200 (77.52%)	198 (76.74%)	0.044	0.834
Yes	59 (16.12%)	113 (33.73%)			58 (22.48%)	60 (23.26%)		
**Anticoagulation**, **no.**(%)								
No	13 (3.55%)	20 (5.63%)	2.28	0.131	13 (5.04%)	14 (5.43%)	0.039	0.843
Yes	353 (96.45%)	315 (94.37%)			245 (94.96%)	244 (94.57%)		
**Duration of surgery (h), no.(%)**								
≤ 2	257 (70.22%)	208 (62.09%)	5.176	0.023	171 (66.28%)	168 (65.12%)	0.077	0.781
>2	109 (29.78%)	127 (37.91%)			87 (33.72%)	90 (34.88%)		
**Time from trauma to surgery(d), no.(%)**								
≤ 7	142 (38.80)	88 (26.27%)	12.455	<0.001	126 (48.84%)	117 (45.35%)	0.63	0.427
>7	224 (61.20)	247 (73.73%)			132 (51.16%)	141 (54.65%)		
**Blood transfusion, no.(%)**								
No	179 (48.91%)	196 (58.51%)	6.48	0.011	136 (52.17%)	117 (45.35%)	0.07	0.791
Yes	187 (51.09%)	139 (41.49%)			132 (51.16%)	141 (54.65%)		
***Test indicators***								
WBC^a^(10^9/L)	8.69 ± 2.96	8.08 ± 2.81	2.8	0.005	8.12 ± 2.63	8.22 ± 2.86	0.405	0.685
PT^a^ (s)	13.51 ± 1.51	13.86 ± 1.65	2.918	0.002	13.52 ± 1.24	13.65 ± 1.29	1.192	0.234
APTT^a^ (s)	36.67 ± 5.19	36.29 ± 6.52	1.387	0.166	36.76 ± 5.07	36.85 ± 6.57	0.163	0.871
PLT^a^ (10^9/L)	167	168.5	0.069	0.945	170	169.50	0.717	0.473
	(109.70,213.25)	(134.00,213.00)			(135.00,213.25)	(125.75,214.00)		
Crea^a^ (mmol/L)	67	66	0.473	0.636	66	66	0.386	0.7
	(47.70,78.00)	(47.60,83.00)			(55.00,79.00)	(56.00,81.25)		
NEUT%^a^ (%)	75.45	75	0.478	0.633	74.4	75.1	1.44	0.15
	(63.37,80.93)	(61.00,80.80)			(68.23,80.15)	(70.10,80.13)		

^a^The inspection index upon admission

**Fig. 2:**
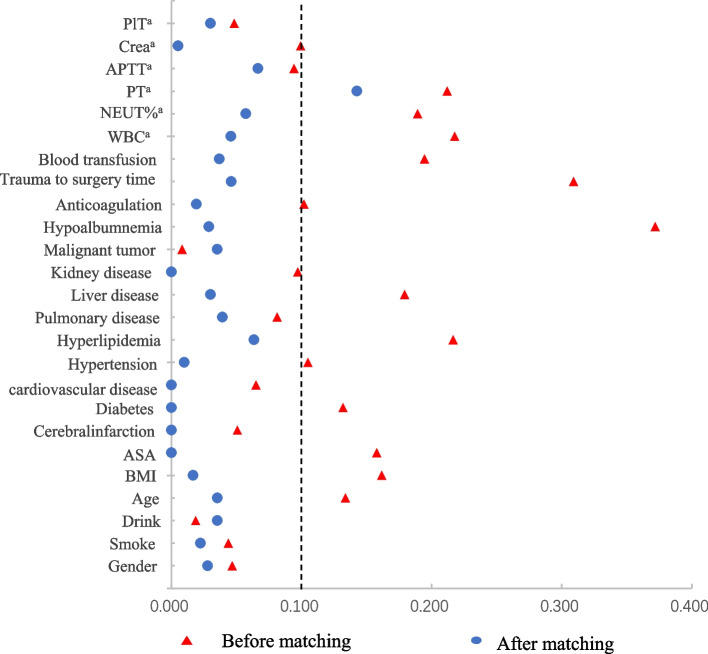
Matching effect and balance test of the two groups before and after matching. Variable standardization difference diagram. ^a^The inspection index upon admission

Equilibrium of the two covariables before and after matching. The standardized difference of each covariable between the two groups before and after matching was calculated (d). Before matching, the standardized values of 11 covariates such as sex, smoking history, drinking history, cerebral infarction, and pulmonary disease were observed between the two groups (d > 0.1). After matching, the d values of 24 covariates were all < 0.1, except for preoperative prothrombin time (PT) level (d = 0.142). This indicates that PSM had a good matching effect, as shown in Fig. 2.

### Incidence of perioperative DVT (Table 3)

Table 3 presents the incidence of perioperative DVT. The total incidence of DVT was 42.35% (379/895); 194 patients had thrombosis before surgery; and 185 patients had thrombosis after surgery, of which 60 patients had thrombosis that progressed after surgery, and 125 patients had newly formed thrombus after operation. The incidence of pulmonary embolism was 1.68% (15/895). Among 15 cases of PE, 44.67% were complicated with distal thrombus, 13.33% (2/15) were complicated with proximal thrombus, and 40% (6/15) were complicated with mixed thrombus.

**Table 3:** Incidence of perioperative deep vein thrombosis in patients with bone trauma (cases %)

The formation of DVT				Fracture side					
The total incidence	Pre-operation	Post-operation	PE	Femoral neck	Femoral intertrochanteric	Femoral shaft	Pelvis	Acetabulum	Multiple fractures
(379) 42.35	(194) 29.16	(185) 20.67	(15) 1.68	360	262	107 (11.96)	100 (11.17)	35 (3.91)	31 (3.46)
				(40.22)	(29.27)				

### Characteristics of the included population (Fig. 3)

**Fig. 3:**
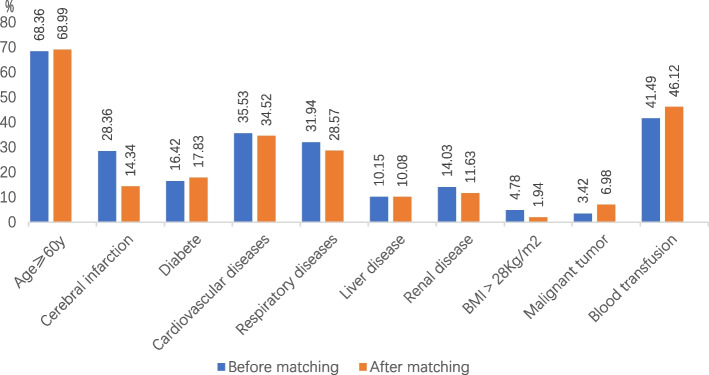
Comparison of anemia patients before and after PSM

Among the 701 patients finally included, 272 (38.80%) were men, and 429 (61.20%) were women; the average age was 68.6 ± 19.9 years. There were 295 femoral neck fractures, 202 intertrochanteric fractures, 75 femoral shaft fractures, 82 pelvic fractures, 23 acetabular fractures, and 24 multiple fractures. Among them, 335 (47.79%) patients had anemia, and 366 (52.21%) did not have anemia. In the anemic group, there were 229 patients (68.36%) aged > 60 years and 95 patients (28.36%) with cerebral infarction. Fifty-five (16.42%) patients were diabetic, 119 (35.52%) had cardiovascular diseases, 107 (31.94%) had respiratory diseases, 34 (10.15%) had hepatobiliary diseases, 47 (14.03%) had renal diseases, 16 (4.78%) were obese (BMI > 28), 24 patients had a malignant tumor (3.42%), and 139 patients had perioperative blood transfusion (41.49%). Among the 24 patients with malignant tumors, 7 patients had breast cancer, 5 had lung cancer, 5 had gastrointestinal tumors (pancreatic cancer, rectal cancer, stomach cancer), 1 patient had thyroid cancer, 3 had urinary system tumors (bladder cancer, prostate cancer), 2 had esophageal cancer, and 1 patient had hematologic system tumors. Among them, 23 people underwent radical surgical treatment, and all of them were regularly reviewed after surgery. Nineteen patients did not relapse after surgery and were generally in good condition, and 4 relapsed after surgery and underwent chemotherapy and immunotherapy; 1 patient received targeted drug therapy and was in stable condition. Among the five patients with lung cancer, two had new postoperative onset of DVT. Patients in the anemic group were older and had a relatively high frequency of perioperative red blood cell transfusion (Fig. 3).

After matching, there were 258 cases in the anemic group and 258 cases in the non-anemic group. There were 178 patients (68.99%) aged > 60 years, 37 (14.34%) with cerebral infarction, 46 with diabetes mellitus (17.83%), 87 with cardiovascular disease (34.52%), 72 with respiratory diseases (28.57%), 26 with hepatobiliary system disease (10.08%), 30 with kidney disease (11.63%), 5 obese (BMI > 28) patients (1.94%), 18 with a malignant tumor (6.98%), and 119 (46.12%) with perioperative transfusion. After matching, rates of anemia were higher among older patients and those with cardiovascular disease and blood transfusion.

### Correlation analysis between preoperative anemia before and after PSM and perioperative DVT (Table 4)

Before PSM, preoperative anemia was statistically significant for the formation of DVT in patients with perioperative bone trauma (*P* < 0.001, RR =1.567 [95% CI 1.217–2.017]). After PSM, preoperative anemia increased the risk of DVT in patients with perioperative bone trauma, the difference was still statistically significant (*P* = 0.009, RR =1.500 [95% CI 1.105–2.036]).

**Table 4:** Correlation analysis between preoperative anemia and perioperative DVT before and after PSM

	B	RR	*P*	95%CI
before matching	0.449	1.567	<0.001	1.217–2.017
After matching	0.405	1.500	0.009	1.105–2.036

### Predictive scores for perioperative DVT from preoperative anemia (Fig. 4)

The ROC plot of preoperative anemia versus perioperative DVT was visualized using IBM SPSS software. According to ROC analysis, the threshold for preoperative anemia associated with DVT was 125 g/L and the area under the ROC curve (AUC) was 0.5877 (95% CI 0.5345–0.6408). On this basis, sensitivity and specificity were 89.2 and 30.3%, respectively, with a Youden index of 0.195. In men and women, the preoperative anemia thresholds were 125 g/L and 114 g/L, respectively, and the AUC was 0.5856 (95% CI 0.5001–0.6713) and 0.5975 (95% CI 0.5309–0.6641), respectively.

**Fig. 4:**
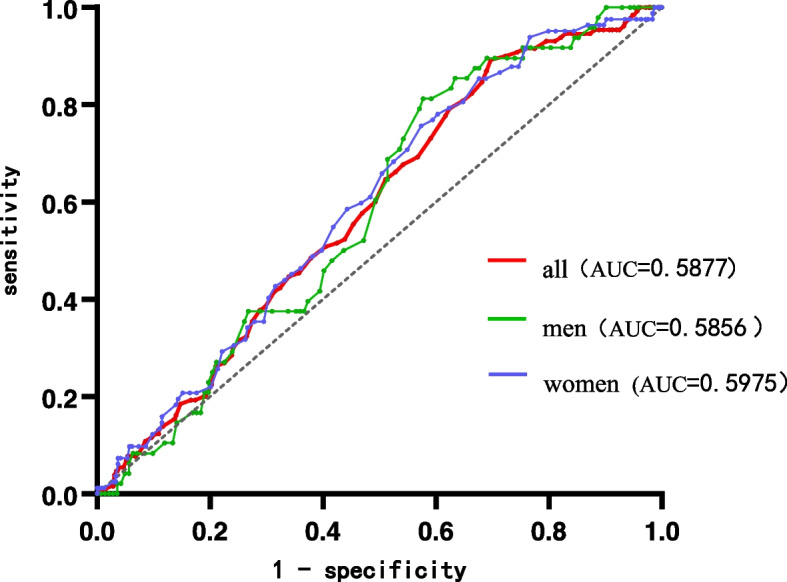
Receiver operating characteristic curves for pre-operative hemoglobin concentration associated with deep vein thrombosis in patients with perioperative bone trauma**.** AUC:Area under the curve

The ROC plot of preoperative anemia versus perioperative DVT was visualized using IBM SPSS software. According to ROC analysis, the threshold for preoperative anemia associated with DVT was 125 g/L and the area under the ROC curve (AUC) was 0.5877 (*P* < 0.001, [95% CI 0.5345–0.6408]). On this basis, sensitivity and specificity were 89.2 and 30.3%, respectively, with a Youden index of 0.195. In men and women, the preoperative anemia thresholds were 125 g/L and 114 g/L, respectively, and the AUC was 0.5856 (*P* = 0.076, [95% CI 0.5001–0.6713]) and 0.5975 (*P* < 0.001,[95% CI 0.5309–0.6641]), respectively. In men, sensitivity and specificity were 81.3 and 42.3%, with a Youden index of 0.235. In women, sensitivity and specificity were 75.6 and 42.6%, respectively, with a Youden index of 0.182.

#### Sensitivity analysis

In the entire cohort (*N* = 701), after adjusting for all the covariates in Table 2, multivariate Poisson regression analysis showed that preoperative anemia was associated with perioperative DVT risk (*P* = 0.018, RR = 1.36 [95% CI 1.05–1.74]). In addition, we adjusted the propensity score, and the Relative Risk (RR) was similar (*P* = 0.009, RR = 1.50 [95% CI 1.11–2.04]) (Table 5). We generated one E-value to assess whether sensitivity to unmeasured confounding was also reliable; our results were considered meaningful unless there was an unmeasured confounding factor whose risk of perioperative DVT was greater than 2.37% or less than 0.43%.

**Table 5:** Associations between the anemic and Nonanemic patient in the crude analysis, multivariable analysis, and propensity-score analyses

Analysis	The incidence of DVT (%)	*P*-value
No. of events/no. of patients at risk (%)		
Non anemia	76/366 (20.77)	
Anemia	109/335 (30.70)	
Crude analysis — hazard ratio (95% CI)	1.56 (1.22–2.02)	< 0.001
Multivariable analysis — hazard ratio (95% CI)^a^	1.36 (1.05–1.74)	0.018
With inverse probability weighting, IPTW^b^	1.63 (1.15–2.29)	0.006
Adjusted for propensity score^c^	1.50 (1.11–2.04)	0.009
With standardized mortality ratio weighting, SMRW^d^	1.8 (1.28–2.51)	0.001

^a^Shown is the hazard ratio from the multivariable logistic regression model, with adjusted for all covariates

^b^Shown is the primary analysis with a hazard ratio from the multivariable logistic regression model with the same strata and covariates with inverse probability weighting according to the propensity score

^c^Shown is the hazard ratio from a multivariable logistic regression model with the same strata and covariates with matching according to the propensity score. The analysis included 258 patients (258 were anemic and 258 were non-anemic before surgery)

^d^hown is the hazard ratio from a multivariable logistic regression model with the same strata and covariates, with additional standardized mortality ratio weighting

## Discussion

This study showed that the overall incidence of postoperative DVT in patients with bone trauma was 20.67%, and the incidence of PE was 1.68%. The incidence of perioperative DVT was higher for orthopedics, especially in patients with bone trauma, with an incidence of DVT after orthopedic major surgery reportedly ranging from 40 to 60% [2, 21–23]. Risk factors associated with DVT in patients with perioperative bone trauma included lower extremity and pelvic fractures, high-energy trauma, Glasgow score, prolonged preoperative waiting time, prolonged surgery time, advanced age, obesity, history of malignancy, preoperative anemia, and preoperative blood transfusion [13, 24–26]. Among these, factors associated with red blood cells such as anemia and blood transfusion are increasingly concerning. Liu et al. found that anemia is an independent risk factor for DVT [11], which is consistent with our findings. We found that preoperative anemia before PSM was an independent risk factor for DVT in patients with perioperative bone trauma (RR 1.567, 95% CI 1.217–2.017, *P* < 0.001). After further matching the propensity scores of 25 confounding factors such as preoperative comorbidities, preoperative test indicators, and perioperative blood transfusion, we found that this correlation was still valid (RR =1.500, 95% CI 1.105–2.036, *P* = 0.009), and the risk of preoperative anemia was 0.5 times higher than that of normal patients, which verified our conclusion that preoperative anemia was the cause of DVT in patients with perioperative bone trauma.

In this study, we found that the incidence of anemia in patients with bone trauma was 47.09%(494/1049), ranging from 29.74% of hemoglobin (Hb) levels below 10 g/dL to 63.30% of hemoglobin levels below 12 g/dL, and postoperative anemia rates as high as 84.94% (894/1049). Studies have shown that the incidence of preoperative anemia in patients undergoing orthopedic surgery is 12.8–24.3% for hip and knee arthroplasty [27.28], 21–24% for spinal elective surgery [27, 28], and 42–45% for trauma orthopedic surgery [24, 29, 30]. Patients with hip fracture may experience a preoperative Hb decline of more than 20 g/L [31], with an average decrease of 30 g/L and a 45% transfusion rate after surgery [32]. The incidence of anemia after hip and knee replacement is more than 80% [28, 32], the rate of anemia after spinal surgery is 82.7 to 85.8% [27, 29], and the rate of anemia after bone tumor surgery is 89.2% [33]. Zhou et al. analyzed 20,308 patients in China showing that the incidence of anemia before total hip arthroplasty was 26.1%, that before total knee replacement (TKA) was 25.5%, and that before femoral head replacement was 43.9%; the incidence of postoperative anemia was 89.1%, that of TKA was 83.9%, and that of femoral head replacement was 81.9% [34]. Perioperative anemia is common in orthopedic patients. If a patient’s anemia cannot be corrected in time before surgery, the operation itself will cause ischemia and hypoxia of cells, tissues, and organs; increase the occurrence of perioperative complications such as DVT; increase the rate of blood transfusion; increase the infection risk, disability rate, and mortality; delay the recovery of postoperative function; and prolong the length of hospital stay [31].

The causes of preoperative anemia are roughly as follows: 1) hemorrhagic anemia caused by traumatic fractures in patients with bone trauma, bleeding during surgery, anemia caused by malnutrition in elderly patients; and 2) anemia caused by the destruction of red blood cells under trauma, inflammation, and stress conditions [35].

In recent years, some studies have analyzed the pathogenesis of anemia promoting thrombosis, which is roughly as follows. Anemia stimulates platelet production, the increase in plasminogen activator inhibitors in anemia decreases the fibrinolytic activity, and the increase in absolute stress of the lining of blood vessels in anemia leads to endothelial damage, resulting in anemia and other mechanisms, resulting in an increased risk of thrombosis in patients with anemia [36–40]. More importantly, the destruction of red blood cells caused by trauma and inflammatory response in patients with bone trauma and exposure of the inner membrane of red blood cells lead to hypercoagulable blood, a thrombosis mechanism based on the red blood cell pathway, which has received increasingly more attention in recent years [7].

Finally, we estimated that the preoperative Hb concentration threshold for perioperative DVT was 125 g/L, the AUC was 0.5877, and the sensitivity and specificity were 87.8 and 37.8%, respectively. Our estimated AUC was slightly lower, which indicates that there are multiple causes of perioperative DVT but also suggests that preoperative Hb has some predictive value for the occurrence of perioperative DVT. We estimated the preoperative Hb thresholds for men and women, which were 127 g/L and 115 g/L, respectively, and the AUCs were 0.5856 and 0.5975, respectively. This implied the importance of identifying high-risk groups of DVT in the early preoperative period and timely intervention to reduce the incidence of perioperative DVT.

Currently, clinical drug prevention is used for DVT, anticoagulation for the coagulation cascade pathway including low-molecular-weight heparin, and oral factor X inhibitors, and antiplatelet drugs for the platelet pathway, such as aspirin. The formation mechanism of perioperative DVT in patients with bone trauma based on the red blood cell pathway offers another possibility for the clinical prevention of DVT, that is, the anticoagulation approach for the red blood cell pathway. For anticoagulation of the red blood cell pathway, or for anemia that exists before surgery, it is clinically necessary to actively deal with the inflammatory and stress state caused by trauma, reduce the risk of red blood cell destruction, and administer corresponding nutritional support, iron supplementation, and reasonable perioperative blood management to avoid unnecessary red blood cell transfusion. Reducing the incidence of intraoperative anemia and strengthening intraoperative transfusion management, such as with the use of tourniquets, intraoperative controlled antihypertensives, and the use of antifibrinolytic drugs (tranexamic acid), can safely and effectively reduce the need for perioperative allogeneic blood transfusions in orthopedic surgery [41, 42]. For the prevention and treatment of postoperative anemia, the reduction of postoperative bleeding, compression bandaging, and ice packing at the orthopedic incision site, postoperative nutritional support, and iron supplementation can effectively reduce the incidence of postoperative anemia. It is worth noting that blood transfusion and use of Erythropoietin will increase the incidence of perioperative DVT [15–17].

For anticoagulation of the erythrocyte pathway, in addition to preventing and treating perioperative anemia and further optimizing blood products, it is possible to reduce venous thrombosis by restricting erythrocyte incorporation, which may be a novel therapeutic target for reducing venous thrombosis. Rencently, some studies have found that red blood cells can modify the composition of venous thrombosis, which includes fibrin and red blood cells [43–45]. Among them, the activity of the transglutaminase factor XIII (FXIII) is critical crucial for RBC retention within clots and directly affects the size of the thrombus. This indicates that RBCs are not simply trapped by the fibrin meshwork during thrombus formation, but that once present, they must be actively retained within the thrombus through this enzyme activity during clot retraction. Thus, in the anticoagulant pathway against the erythrocyte pathway, drugs that can prevent or reduce the size of the thrombus may be an attractive thromboprophylaxis.

Hemoglobin level can be regarded as comprehensive manifestation of a variety of pathophysiologies in acute or chronic diseases, and the destruction of red blood cells in patients with bone trauma may also be a pathogenic pathway of thrombosis, such as via trauma, inflammation, and other stress effects, as well as weakness, malnutrition, and chronic inflammation. In these cases, the body’s immune imbalance and the production of many cytokines have a certain degree of influence on the number and lifespan of red blood cells. Clinically, the measurement of hemoglobin concentration is more extensive and less costly, and is a routine indicator of preoperative examination. In this study, we found that the Hb concentration was associated with DVT for perioperative bone trauma, regardless of whether Hb was directly related to thromboembolism. Clinicians should pay attention to this preoperative test to avoid the associated risks.

The formation of DVT is a complex process that, in addition to the classical Virchow triad is influenced by many unknown factors. Compared with most previous studies of perioperative DVT risk factors in patients with bone trauma, in this study, we used PSM and modified Poisson regression analysis to explore the correlation between preoperative anemia and perioperative DVT in patients with femoral and pelvic fractures. This reduced the confounding bias in the study and achieved a balanced distribution of covariates between case groups and control groups to achieve effects similar to those of prospective randomized controlled trials. We further analyzed the sensitivity and estimated the E-value, which verified the robustness of our results. In summary, we conducted an observational cohort study to explore the causal relationship between preoperative anemia and perioperative DVT in patients with bone trauma, which has not been reported in previous studies.

### Limitations

This study has multiple limitations. First, this was a single-center retrospective study in which data collection may be influenced by selection bias and other factors. Although we used PSM to control bias caused by 25 confounding factors, the potential for residual confounding exists, as with all retrospective analyses. We used E-value sensitivity analysis to quantify the potential implications of unmeasured confounders and found that an unmeasured confounder was unlikely to explain the entirety of the treatment effect. Second, we set different hemoglobin level thresholds based on sex. Defining the hemoglobin level for anemia is not an absolute value and may vary depending on the different clinical situations. Third, we did not do further analysis based on the severity of anemia and were unable to determine the relationship between the degree of anemia and DVT formation. This may require follow-up studies to clarify further.

## Conclusions

Preoperative anemia is one cause of DVT in patients with perioperative bone trauma. For preoperative anemia in patients with bone trauma surgery, attention is needed to the perioperative stage DVT screening. In patients with bone trauma and complicated DVT, in addition to the coagulation cascade pathway and platelet pathway, the erythrocyte pathway may be a potential pathogenic pathway; however, this requires further study.

## Data Availability

All the data will be available upon motivated request to the corresponding author of the present paper.

## Abbreviations

VTE: Venous thromboembolism
DVT: Deep vein thrombosis
PE: Pulmonary embolism
BMI: Body mass index
ASA: American Society of Anesthesiologists
Crea: Creatinine
PT: Prothrombin time
APTT: Activated partial thromboplastin time
WBC: White blood cell
Hb: Hemoglobin
PLT: Platelet count
NEUT%: Neutrophil percentage
AUC: Area under the curve
